# Supplementary material for: Effect of Fibroblast Growth Factor 21 on the Development of Atheromatous Plaque and Lipid Metabolic Profiles in an Atherosclerosis-Prone Mouse Model
Source: Int J Mol Sci. 2020 Sep 17;21(18):6836. doi: 10.3390/ijms21186836 (PMC7555741; doi:10.3390/ijms21186836)
Supplement: Supplementary file 1 [file ijms-21-06836-s001.pdf]

International Journal of
Molecular Sciences

MDPI

Supplementary Materials

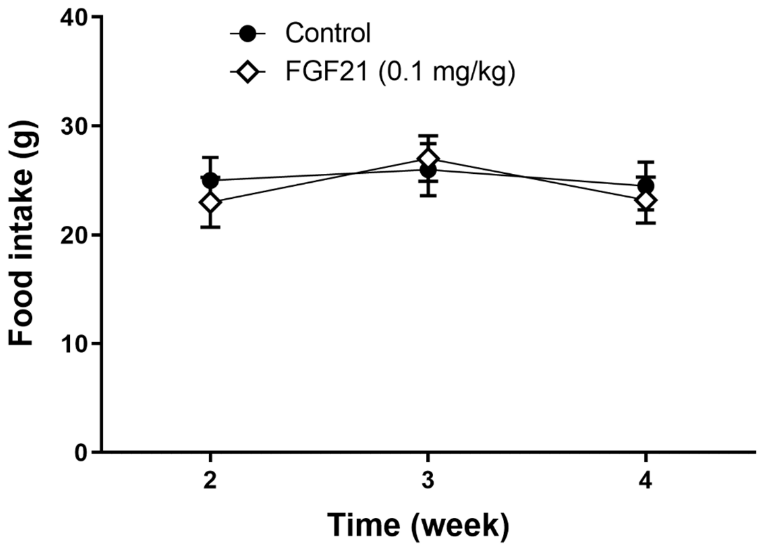

Figure S1. Food consumption during the study period. There was no difference in amount of food consumption between the two groups. FGF21 (fibroblast growth factor 21).

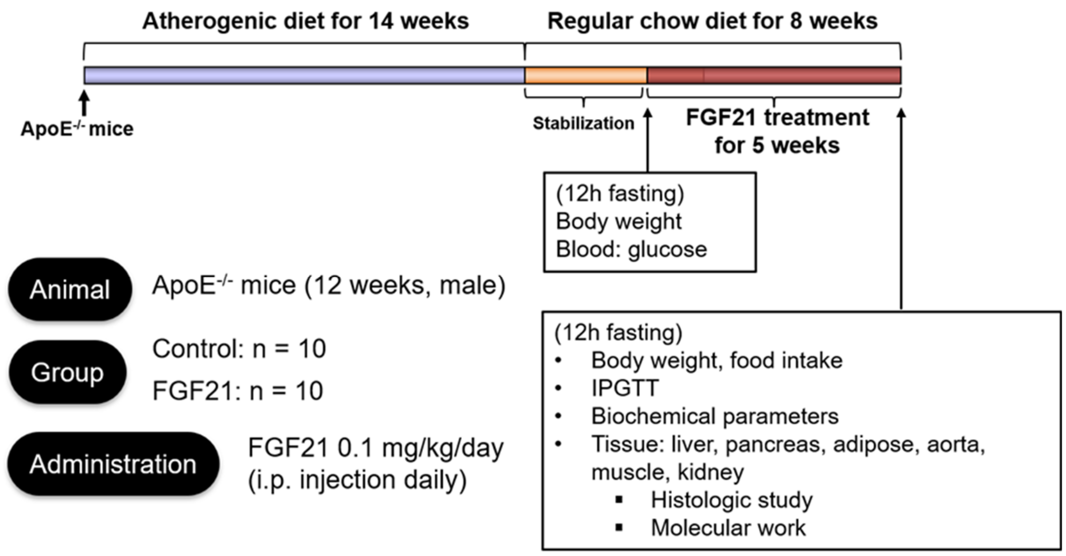

Figure S2. Study design. FGF21 (fibroblast growth factor 21), IPGTT (intraperitoneal glucose tolerance test).
